# Evaluation of the effectiveness of care groups in expanding population coverage of Key child survival interventions and reducing under-5 mortality: a comparative analysis using the lives saved tool (LiST)

**DOI:** 10.1186/s12889-015-2187-2

**Published:** 2015-09-02

**Authors:** Christine Marie George, Emilia Vignola, Jim Ricca, Tom Davis, Jamie Perin, Yvonne Tam, Henry Perry

**Affiliations:** Department of International Health, Program in Global Disease Epidemiology and Control, Johns Hopkins Bloomberg School of Public Health, 615 N. Wolfe Street, Room E5535, Baltimore, MD 21205-2103 USA; Jhpiego, Maternal Child Health Integrated Program (MCHIP), Washington, DC USA; Food for the Hungry, Hunger Corps, Phoenix, Arizona USA

## Abstract

**Background:**

Globally, less than half of Countdown Countries will achieve the Millennium Development Goal of reducing the under-5 mortality rate (U5MR) by two-thirds by 2015. There is growing interest in community-based delivery mechanisms to help accelerate progress. One promising approach is the use of a form of participatory mothers’ groups, called Care Groups, for expanding coverage of key child survival interventions, an essential feature for achieving mortality impact.

**Methods:**

In this study we evaluate the effectiveness of Care Group projects conducted in 5 countries in Africa and Asia in comparison to other United States Agency for International Development-funded child survival projects in terms of increasing coverage of key child survival interventions and reducing U5MR (estimated using the Lives Saved Tool, or LiST). Ten Care Group and nine non-Care Group projects were matched by country and year of program implementation.

**Results:**

In Care Group project areas, coverage increases were more than double those in non-Care Group project areas for key child survival interventions (*p* = 0.0007). The mean annual percent change in U5MR modelled in LiST for the Care Group and non-Care Group projects was −4.80 % and −3.14 %, respectively (*p* = 0.09).

**Conclusions:**

Our findings suggest that Care Groups may provide a promising approach to significantly increase key child survival interventions and increase reductions in U5MR. Evaluations of child survival programs should be a top priority in global health to build a greater evidence base for effective approaches for program delivery.

## Background

The under-5 mortality rate in the least-developed countries of the world has declined from 171 to 98 deaths per 1000 live births between 1990 and 2011 [1]. Despite this progress and the highly effective and inexpensive community-based interventions for addressing the leading causes of under-5 mortality now available [2, 3], less than half of the 75 Countdown Countries will achieve the Millennium Development Goal (MDG) for children of reducing the under-5 mortality rate by two-thirds by 2015 [4]. Furthermore, to achieve the new post-MDG target of ending preventable child deaths by 2015 [5], the annual rate of decline in under-5 mortality will need to double [6].

The child survival literature is replete with studies of intervention effectiveness, analysis of constraints encountered in program implementation, and policy issues. One of the important gaps in the literature, however, is identifying effective delivery strategies for expanding coverage of key child survival interventions. The need for studies on effective delivery strategies is urgent-among the Countdown countries, median national coverage of almost all of the key child survival interventions apart from immunizations and Vitamin A is less than 50 % [4].

The United States Agency for International Development (USAID) has been funding non-governmental organizations (NGOs) to implement child survival programs through the Child Survival and Health Grants Program (CSHGP) since 1987. CSHGP projects implement a standard set of proven high-impact technical interventions and work collaboratively with Ministries of Health and communities to promote behavior change and increased health service utilization through frequent contact with community leaders, groups of mothers, and household visits [7]. One analysis of 12 CSHGP-supported child survival projects provided plausible evidence that these projects doubled the rate of decline in under-5 mortality relative to that in other areas in the same country where the projects were not being implemented (5.8 % versus 2.5 %) [8].

There is a lack of standardized and rigorously evaluated strategies for delivery of community-based child survival interventions. In this study we evaluate Care Groups, a community-based delivery strategy that has emerged through CSHGP, in comparison to other CSHGP-funded child survival programs which do not utilize a common standardized strategy, in terms of increasing coverage of key child survival interventions and reducing under age five mortality, using mortality estimates from the Lives Saved Tool (LiST).

### The care group model

The Care Group model was developed by World Relief, an international NGO based in Baltimore, MD, and its child survival staff working in Gaza Province, Mozambique in the late 1990s. The approach involves the formation of mothers’ groups of approximately 10 Care Group volunteers who are each responsible for visiting on average 10 households closest to their home. A facilitator (i.e., supervisor) visits a Care Group every 2–4 weeks to teach the volunteers 1–3 new key messages to share with their neighbors. Household visits by Care Group volunteers are conducted every 2 weeks. Over a 2-year period, an array of lessons covering household behaviors to promote maternal and child health and health-care utilization are disseminated to every household in the project area where there is a pregnant woman or a mother of a child less than 59 months of age. Through the use of volunteers at the household level, rather than paid promoters, the Care Group model presents a low cost approach for program delivery. One paid local facilitator (often called a promoter) can oversee the activities of approximately 400–1000 households [9–11].

To date, 23 NGOs have implemented the Care Group Model in 27 countries [12, 13]. Two peer-reviewed publications have documented the effectiveness of the Care Group Model in increasing coverage of child survival interventions and reducing under age five mortality [14–16]. However, none of these studies have conducted a systematic evaluation of Care Group projects compared to other child survival programs.

### Features of the care group and Non-care group projects included in the analysis

Below we describe the typical delivery approaches used for Care Group projects compared to other CSHGP child survival projects, non-Care Group projects, through two representative examples. The non-Care Group projects all use collaborative approaches with communities and community groups to expand coverage of key child survival interventions and most utilize home visitation in some form along with women’s group meetings (e.g., mothers clubs). However, they vary widely in the number of home visits per month and the form of women’s groups utilized.

#### World relief/Rwanda care group project, 2001–2006

World Relief implemented a Care Group project in Kibogora district (now part of Nyamasheke District) in Cyangugu Province of Rwanda between 2001 and 2006. The Rwandan Ministry of Health, the Kibogora Hospital, local leaders, church representatives, and local health facility workers were partners in implementing trainings and project activities. The interventions included: immunization promotion, nutrition education, education about the prevention and treatment of diarrhea and malaria, education about maternal and newborn care, and awareness and prevention of sexually transmitted diseases (STIs) and HIV/AIDS. The target population consisted of 24,021 children 0–59 months of age and 35,798 women 15–49 years of age. Interventions were implemented at the community level by Care Group Volunteers and at the health center level by health facility workers. Behavior change strategies included interpersonal communication, support groups, and peer communication. The project recruited more than 2800 volunteers who were organized into 233 Care Groups.

#### Concern worldwide/Rwanda Non-care group project 2001–2006

Concern Worldwide implemented a non-Care Group child survival project in Kibilizi Health District of Butare Province (now part of Gisagara District) in Rwanda between 2001 and 2006. The project was implemented in partnership with the Rwandan Ministry of Health and its district health management team. The target population consisted of 24,494 children 0–59 months and 35,599 women 15–49 years of age.

Project interventions included education about the prevention and treatment of malaria, nutrition education, education about maternal and newborn care, and awareness and prevention of sexually transmitted diseases (STIs) and HIV/AIDS. The project included the training and participation of traditional healers, health facility workers, volunteer CHWs and community-based organizations. Behavior change strategies included mass media campaigns, interpersonal and peer communication, and support groups. The project worked with 479 existing volunteer CHW who were members of 12 associations (4 CHW associations, 4 persons living with HIV/AIDS associations, and 4 traditional birth attendant associations).

## Methods

### Study inclusion criteria

A list of Care Group projects funded by the CSHGP was obtained from the Care Group Info website [17] in March 2013. Information on non-Care Group projects, including the data from the baseline and endline household surveys, was obtained from the USAID Evaluations database [18].

Care Group projects were selected for inclusion in the present analysis if the following eligibility criteria were met:

1. There was a Demographic Health Survey (DHS) or Multiple Indicator Cluster Survey (MICS) available for the country where the Care Group project was implemented within 3 years of both the project baseline and endline household surveys.
2. There was at least one non-Care Group child survival project funded by CSHGP that was implemented in the same country within 3 years of the Care Group project that met criterion 1. This allowed us to match Care Groups and non-Care Groups by country.
3. The project had baseline and endline Knowledge, Practices, and Coverage (KPC) data for a clearly defined subnational area in which it was intervening.

Ten Care Group and nine non-Care Group child survival projects met these study eligibility criteria. Six of the projects took place in Cambodia, three in Kenya, three in Malawi, four in Mozambique, and three in Rwanda (Table 1). All of these projects were implemented between 1998 and 2010, and the majority lasted 5–6 years.

**Table 1:** **C**haracteristics of care group and non-care group child survival projects included in the analysis by country

Country	Region	NGO	Type	Project Period	Estimated Children 0–59 Months in Project Area	Number of the 17 High-impact interventions^a^	Project Cost USD
Cambodia	Kampong Thum	Adventist Development Relief Agency (ADRA)	Non-Care Group	2001–2006	17,477	11	$1,891,059
Cambodia	Battambang	Catholic Relief Services (CRS)	Non-Care Group	2001–2006	24,896	10	$2,023,057
Cambodia	Kampong Chhnang	International Relief and Development (IRD)	Non-Care Group	2006–2010	6217	12	$1,749,948
Cambodia	Siem Reap	Red Cross	Care Group	2005–2008	43,610	12	$2,000,096
Cambodia	Kompong Cham	World Relief (WR)	Care Group	1998–2002	12,167	7	$1,333,376
Cambodia	Kompong Cham	World Relief (WR)	Care Group	2003–2007	12,875	8	$1,583,376
Kenya	Western Province	African Medical and Research Foundation (AMREF)	Non-Care Group	2005–2010	31,644	6	$2,139,312
Kenya	Rift Valley	HealthRight	Non-Care Group	2006–2010	48,844	7	$2,249,261
Kenya	Coast	Plan International	Care Group	2004–2009	46,354	11	$2,000,000
Malawi	Southern Region	International Eye Foundation	Non-Care Group	2002–2006	42,500	7	$2,195,478
Malawi	Northern Region	World Relief (WR)	Care Group	2000–2004	36,732	5	$1,333,331
Malawi	Northern Region	World Relief (WR)	Care Group	2005–2009	32,025	11	$2,022,034
Mozambique	Sofala	Food for the Hungry (FH)	Care Group	2006–2010	60,666	9	$2,026,191
Mozambique	Sofala	Food for the Hungry (FH)	Care Group	2009–2010	83,778	9	$997,975
Mozambique	Manica and Sofala Provinces	Health Alliance International (HAI)	Non-Care Group	2002–2007	97,200	5	$2,000,425
Mozambique	Gaza Province	World Relief (WR)	Care Group	2004–2009	33,451	10	$3,333,333
Rwanda	Butare Province	Concern Worldwide	Non-Care Group	2001–2006	24,494	10	$1,854,243
Rwanda	Kibungo	International Rescue Committee (IRC)	Non-Care Group	2001–2005	109,700	5	$2,004,134
Rwanda	Cyangugu	World Relief (WR)	Care Group	2001–2006	24,021	10	$1,933,657

^a^Number of the 17 High-impact interventions included in LiST for which baseline and endline coverage levels were available

### LiST modeling

The Lives Saved Tool (LiST) is a modeling tool that estimates the impact on mortality outcomes of scaling up 30 child health interventions [19]. LiST-specified effect sizes for each intervention are derived according to standards set by from the Child Health Epidemiology Reference Group [20]. In the present study, LiST version 4.68 was used to estimate the impact of each child survival project on the under-5 mortality rate (U5MR). This version of the software was downloaded from the Johns Hopkins Institute for International Programs website in March 2013 [21]. The methods and assumptions of the LiST tool have been previously described [22]. LiST has been validated to produce reasonably accurate estimates of national under-5 and neonatal mortality, when coverage changes and other necessary parameters are accurately measured [23]. LiST has also been validated to produce reasonable sub-national mortality estimates in various settings in South Asia and Africa [24–28].

We modeled baseline mortality rates for child survival project areas using subnational DHS or MICS mortality data (Table 2). Since DHS and MICS subnational under-5 mortality rates are derived from deaths that occurred over a 10-year period prior to the survey, we used mortality data from the later of the two DHS or MICS reports as the baseline mortality rate modeled in LiST. This allowed us to have a more recent estimate of U5MR in the population. DHS reports were downloaded from the Measure DHS website [2]. MICS reports were obtained from UNICEF’s Child Info website [16].

**Table 2:** Estimated annual percent change in under-5 mortality rate (u5mr) in project areas modelled in list

Country	Region	Organization	Project Type	Project Period	DHS Survey^a^	Estimated Annual Percent Change in U5MR for Project Area
Cambodia	Kampong Thum	ADRA	Non-Care Group	2001–2006	2005–2010	−2.54 %
Cambodia	Battam-bang	CRS	Non-Care Group	2001–2006	2005–2010	−5.71 %
Cambodia	Kampong Chhnang	IRD	Non-Care Group	2006–2010	2005–2010	−4.45 %
Cambodia	Siem Reap	RC	Care Group	2005–2008	2005–2010	−5.99 %
Cambodia	Kompong Cham	WR	Care Group	1998–2002	2000–2005	−3.40 %
Cambodia	Kompong Cham	WR	Care Group	2003–2007	2005–2010	−7.16 %
Kenya	Western Province	AMREF	Non-Care Group	2005–2010	2003–2009	−2.86 %
Kenya	Rift Valley	HealthRight	Non-Care Group	2006–2010	2003–2009	−3.56 %
Kenya	Coast	Plan	Care Group	2004–2009	2003–2009	−3.78 %
Malawi	Southern Region	IEF	Non-Care Group	2002–2006	2004–2010	−3.64 %
Malawi	Northern Region	WR	Care Group	2000–2004	2004–2010	−2.02 %
Malawi	Northern Region	WR	Care Group	2005–2009	2004–2010	−4.43 %
Mozambique	Sofala (Area A)	FH	Care Group	2006–2010	2008–2011	−3.82 %
Mozambique	Sofala (Area B)	FH	Care Group	2009–2010	2008–2011	−8.48 %
Mozambique	Manica and Sofala	HAI	Non-Care Group	2002–2007	2008–2011	−3.66 %
Mozambique	Gaza Province	WR	Care Group	2004–2009	2008–2011	−3.24 %
Rwanda	Southern Region	Concern	Non-Care Group	2001–2006	2005–2008	−1.95 %
Rwanda	Eastern Region	IRC	Non-Care Group	2001–2005	2005–2008	0.08 %
Rwanda	Western Region	WR	Care Group	2001–2006	2005–2008	−5.70 %

^a^Demographic and Health Survey (DHS) used for Baseline Mortality and Supplemental Data

### Modeling project coverage changes in LiST

Project coverage data at baseline and endline were obtained from either the project household KPC surveys (obtained from the USAID CSHGP or Care Group Info website) or from project staff [18]. The methodology used for KPC survey sampling is described elsewhere [8]. All USAID CSHGP-funded organizations are required to report the results of their KPC surveys using a standard methodology in their final evaluation reports, and these reports are uploaded to the USAID Evaluations web-based database [29]. If project coverage data were not available for specific indicators at baseline or endline, they were supplemented by DHS or MICS coverage data for the subnational region where the project was implemented. The proportion of the subnational population covered by the projects ranged from 3 to 100 % with a median of 16 %. The International Rescue Committee project in Kibungo Province, Rwanda conducted between 2001 through 2005 targeted the entire subnational population.

In our analysis we focus on the 17 high-impact coverage indicators modeled in LiST that were evaluated in a similar analysis conducted by Ricca et al. (Table 3) [8]. If the exact LiST indicator was not available in the project report or DHS surveys, we used a similar “project indicator” described in Table 3, based on previous methods implemented in Ricca et al. [8].

**Table 3:** Summary table of high-impact coverage indicators modelled in LiST

Indicator	LiST indicator	LiST indicator description	Standard CSHGP Project indicator description
ANC4	4 or more antenatal care visits (ANC4+)	Percentage of women who attended 4 or more antenatal care visits during their most recent pregnancy	Percentage of women who had a live birth in the 5 years preceding the survey who had 4 or more antenatal care visits during their most recent pregnancy
TT2	Tetanus toxoid (TT) vaccination	Percentage of women who received 2 doses of tetanus toxoid during this pregnancy or ever	Percentage of mothers with a child aged 0- < 24 months who received at least 2 tetanus toxoid injections before the birth of their youngest child
IFA	Multiple micronutrient supplementation	Percentage of pregnant women taking a multiple micronutrient supplement daily	Percentage of women with a birth in the 5 years preceding the survey who took iron tablets or syrup for 90 or more days during their most recent pregnancy
IPTp	Pregnant women protected via intermittent treatment of malaria or use of an ITN	Percentage of pregnant women receiving 2+ doses of SP/Fansidar during pregnancy or sleeping under an insecticide treated bed net	Percentage of women who took 2+ doses of SP/Fansidar during pregnancy for last live birth
SBA	Skilled birth attendance	Percentage of children born who were attended by a skilled attendant (such as a doctor, nurse or midwife)^a^	Percentage of children aged 0- < 24 months whose births were attended by skilled health personnel^a^
EBF	Exclusive breast feeding	Percentage of children 0- < 6 months of age receiving only breast milk for food (plus medication, vaccines, and vitamins)	Percentage of children 0- < 6 months of age who were exclusively breastfed during the previous 24 h
Comp Feed	Complementary feeding	Percentage of children 6- < 24 months of age receiving all three ICYF practices: continued breastfeeding, appropriate quantity of foods, and dietary diversity	Percentage of children 6- < 9 months of age receiving breast milk and complementary foods
PPV	Postnatal preventive care	Visit to a health professional (or home visit from a trained health worker) within 2 days of delivery	Percentage of children aged 0- < 24 months who received a post-natal visit from an appropriate trained health worker within 3 days after birth
Vit A	Vitamin A supplementation	Percentage of children 6–59 months of age receiving 2 doses of Vitamin A during the last 12 months	Percentage of children aged 6‐ < 24 months who received a dose of Vitamin A in the last 6 months
ITN	Insecticide-treated bed net	Percentage of households owning at least 1 insecticide-treated bed net or protected by indoor residual spraying	Percentage of children aged 0‐ < 24 months who slept under an insecticide‐treated bed net (in malaria risk areas, where bed net use is effective) the previous night
Meas	Measles	Percentage of children 12- < 24 months who have received 1 dose of measles vaccine	Percentage of children aged 12- < 24 months who have received a measles vaccine
Full Vacc	Full vaccination with EPI vaccines	Percentage of children 12- < 24 months of age who have received 3 doses of DPT vaccine	Percentage of children aged 12–23 months who received 3 doses of DPT vaccine before they reached 12 months
Hand Wash	Hand washing with soap	Percentage of mothers using appropriate hand washing practices including washing hands with soap, ash, or other materials and using adequate water after handling feces and before preparing food	Percentage of caretakers with children aged 0- < 24 months who reported washing hands with soap before food preparation, before child feeding, and after defecation
Latrine	Improved sanitation	Percentage of households with access to improved sanitation	Percentage of households using flush toilet or ventilated improved pit latrine
ORT	Oral rehydration therapy	Percentage of children with diarrhea treated with oral rehydration solution, including sachets or pre-mixed solutions	Percentage of children aged 0- < 24 months with diarrhea in the previous 2 weeks who received oral rehydration solution (ORS) and/or recommended home fluids
Abx Pneum	Oral antibiotics: case management of pneumonia in children	Percentage of children with suspected pneumonia treated with appropriate antibiotics	Percentage of the children aged 0- < 24 months with cough and fast and or difficult breathing during the previous 2 weeks who were taken to an appropriate health provider
Mal Treat	Antimalarial/artemesinin compounds for malaria	Percentage of children treated within 48 h of the onset of fever in malaria endemic areas with an artemesinin containing compound	Percentage of children aged 0- < 24 months with a febrile episode during the previous 2 weeks who were treated with an effective anti-malarial drug within 24 h after the fever began

### Statistical analysis

The U5MR estimated by the LiST tool was used to determine the annual percentage change in U5MR over the project period. We compared changes in intervention coverage and U5MR between the Care Group and non-Care Group CSHGP projects using the Wilcoxon signed-rank test. The mean number of households per CHW for Care Group and non-Care Group CSHGP projects were compared using the signed-rank test. There is some debate in the statistical literature relating to the analysis of matched data as we have, where matching is based on intervention status, and whether matching needs to be explicitly accounted. In our present study we do not match by country cluster because our research question is more focused on the ability of the Care Group child survival programs to change intervention coverage, and reduce annual U5MR rather than differences in project delivery between countries [30].

### Ethical approvals

We obtained an exemption from the Johns Hopkins institutional review board to conduct our analysis.

## Results

### Coverage changes for care group and Non-care group child survival projects

Changes in the 17 high-impact coverage indicators included in the LiST analysis are presented for the 10 Care Group and 9 non-Care Group CSHGP projects in Table 4. On average, each Care Group child survival project implemented 10 out of the 17 high-impact interventions modeled in LiST compared to 7 for the non-Care Group projects. In Care Group project areas, coverage increases for high impact interventions were more than double those in non-Care group project areas for antenatal care visits, tetanus toxoid vaccination, multiple micronutrient supplementation, complementary feeding, hand washing with soap, oral rehydration therapy, oral antibiotics for pneumonia, and malaria treatment. Overall, Care Group projects yielded significantly higher increases in coverage than non-Care Group projects for high-impact coverage indicators measured (*p* = 0.0007).

**Table 4:** Mean change in % (N) population coverage for Care Group and non-Care Group child survival projects by intervention

Type of project	Intervention															
	ANC4	TT2	IFA	IPTp	SBA	EBF	Comp Feed	PPV	Vit A	ITN	Meas	Full Vacc	Hand Wash	ORT	Abx Pneum	Mal Treat
Care Group Projects	29 (1)	25 (9)	67 (3)	35 (3)	27 (7)	44 (9)	22 (3)	-	27 (8)	41 (8)	24 (8)	23 (9)	43 (9)	40 (8)	14 (6)	77 (1)
Non-Care Group Projects	8 (4)	9 (6)	31 (2)	33 (5)	18 (8)	41 (7)	−12 (2)	38 (4)	25 (5)	27 (5)	15 (5)	22 (5)	20 (3)	21 (2)	6 (3)	31 (3)

^a^Does not include trained traditional midwives

The mean number of households per CHW was lower for Care Group projects (11:1) compared to non-Care Group projects (22:1) (*p* = 0.009). The mean expenditure per child life saved was not significantly different between the Care and non-Care Group projects at 62.90 USD and 66.61 USD, respectively.

### LiST modeling

All available coverage indicators were modeled in LiST to calculate the mean percent annual change in U5MR for the Care Group and non-Care Group CSHGP projects. The mean annual percent change in U5MR for the Care Group and non-Care Group projects was −4.80 and −3.14 %, respectively (a ratio of 1.53, *p* = 0.09). The mean annual percent change in U5MR was higher for the Care Group projects versus non-Care Group CSHGP projects for 4 out of the 5 countries included in our analysis.

## Discussion

This analysis presents the first published evaluation of the effectiveness of Care Groups in comparison to other CSHGP-funded child survival programs in terms of increasing coverage of key child survival interventions and estimated reductions in U5MR. When the improvements in coverage of key child survival interventions are compared, Care Group projects had significantly greater increases in coverage for all key interventions, where data was available. Furthermore, for nearly half of the interventions, Care Group projects had twice the increases in coverage observed in the non-Care Group CSHGP projects. When LiST was used to estimate the annual decline in U5MR, Care Group projects had greater reductions in U5MR in four out of the five countries compared to other CSHGP projects. Overall, the decline in U5MR by country was 1.5 times greater for Care Group projects than for other CSHGP projects; however, this finding was not statistically significant (*p* = 0.09). These findings are likely due to the significantly lower ratio of households per community health worker (or volunteer) for the Care Group model compared to the other CSHGP projects (11:1 vs. 22:1), which allowed for greater reinforcement of the key messages disseminated (e.g., >90 % coverage every 2 weeks in Sofala). Consistent with this hypothesis, Care Group projects targeted a greater number of high-impact interventions modeled in LiST compared to non-Care Group projects (mean: 10 vs. 7 interventions). These increases in coverage for the Care Group versus the non-Care Group projects were most striking for multiple micronutrient supplementation (67 vs. 31 %), oral rehydration therapy (41 vs. 20 %), antibiotics for pneumonia (14 vs. 6 %), and insecticide treated nets (41 vs. 27 %), which are high impact interventions demonstrated to reduce U5MR. [8]

Our present analysis has several limitations. First, since we wanted to compare Care Group projects with non-Care Group projects implemented in the same country around the same time (to control for contextual influences), the sample size of our current evaluation was small. This likely limited our power to detect significant differences between Care group and non-Care Group projects in terms of mean annual percent change in U5MR. As more Care Group projects are implemented, a meta-analysis of these projects is urgently needed. Second, there were some differences observed between CSHGP project and LiST definitions for coverage indicators. However, these differences should be consistent between Care Group and non-Care Group projects, and therefore minimally affect comparisons of mortality estimates between the two groups. Third, we lacked data on actual mortality impact for the projects; therefore, we relied on estimates from LiST. With the goal of validating the use of the LiST tool, a recent evaluation of a Care Group project implemented in Mozambique found that the LiST modelling of intervention coverage data, when compared to direct measurements of mortality decline, yielded reasonably accurate estimates of mortality reduction (39 % compared to 34 %, respectively) [13]. Fourth, we lacked a control area in the region where each project was conducted. Randomized controlled trials are needed to rigorously evaluate the impact of the Care Group model on changes in coverage and U5MR. Fifth, we did not compare a single community-based delivery mechanism to the Care Group model; instead, we included all other CSHGP projects implemented at the same time and in the same country. Future studies should compare the Care Group model to other commonly used community-based delivery strategies such as Participatory Learning and Action Groups [31].

Our current findings are consistent with previous evidence that the Care Group model is effective in significantly expanding coverage of key child survival interventions [15, 32, 33] reducing undernutrition [15], reducing childhood diarrhea [32], and lowering under-5 mortality [9, 14]. We believe these positive effects are due to the additional reinforcement Care Groups can provide through their significantly lower community worker to household ratio (11:1), a feature that made Care Groups distinct from the other child survival programs included in this analysis. The lower community worker to household ratio allowed more time for lessons, and motivated community workers to switch to new topics and interventions every few months to keep their audience interested.

All countries in the present study are on the Countdown to 2015 list, and therefore among the 74 countries worldwide where 97 % of the world’s child deaths are occurring [4]. Innovations such as the Care Group Model are needed to expand coverage of key child survival interventions to achieve the 2035 goal of ending preventable child mortality, which will require doubling the global rate of decline in under-5 mortality.

## Conclusions

Our findings suggest that the Care Group model may provide a promising approach to significantly expand key child survival interventions and increase reductions under age 5 mortality which warrants future investigation. In spite of the many limitations of this study, we believe that it is an important contribution to the literature. It assesses evidence from multiple settings and projects regarding the effectiveness of the Care Group model in comparison to other child survival projects in improving delivery of multiple child survival interventions, rather than the efficacy of single interventions, which has been the focus of most research to date. Since evaluations of many Care Group projects in addition to those reviewed here are now publicly available, further analysis of these findings is warranted, including a meta-analysis of the findings from all available evaluations. In addition, new programs using the Care Group model are needed that are carefully documented and combined with rigorous external evaluations. Evaluations of integrated child survival programs of all types in the peer-reviewed scientific literature should be a top priority in global health to build a greater evidence base for effective approaches for program delivery.
